# Transcriptomic Analysis of the Porcine Endometrium during Embryo Implantation

**DOI:** 10.3390/genes6041330

**Published:** 2015-12-21

**Authors:** Haichao Lin, Huaizhong Wang, Yanping Wang, Chang Liu, Cheng Wang, Jianfeng Guo

**Affiliations:** 1 Institute of Animal Science and Veterinary Medicine, Shandong Academy of Agricultural Sciences, Jinan 250100, China; E-Mails: marslhc@126.com (H.L.); whzh0825@163.com (H.W.); wangyanping03@163.com (Y.W.); liuchangjlau16@126.com (C.L.); jinanwangcheng@163.com (C.W.); 2 Key Laboratory of Disease Control and Animal Breeding of Shandong Province, Jinan 250100, China

**Keywords:** embryo implantation, endometrium, pigs, pregnancy, sows, transcriptome

## Abstract

In pigs, successful embryo implantation is an important guarantee for producing litter size, and early embryonic loss occurring on day 12–30 of gestation critically affects the potential litter size. The implantation process is regulated by the expression of numerous genes, so comprehensive analysis of the endometrium is necessary. In this study, RNA sequencing (RNA-Seq) technology is used to analyze endometrial tissues during early pregnancy. We investigated the changes of gene expression between three stages (day 12, 18, and 25) by multiple comparisons. There were 1557, 8951, and 2345 differentially expressed genes (DEGs) revealed between the different periods of implantation. We selected several genes for validation by the use of quantitative real-time RT-PCR. Bioinformatic analysis of differentially expressed genes in the endometrium revealed a number of biological processes and pathways potentially involved in embryo implantation in the pig, most noticeably cell proliferation, regulation of immune response, interaction of cytokine-cytokine receptors, and cell adhesion. These results showed that specific gene expression patterns reflect the different functions of the endometrium in three stages (maternal recognition, conceptus attachment, and embryo implantation). This study identified comprehensive transcriptomic profile in the porcine endometrium and thus could be a foundation for targeted studies of genes and pathways potentially involved in abnormal endometrial receptivity and embryo loss in early pregnancy.

## 1. Introduction

Endometrial receptivity is critical for the establishment of successful implantation; this depends on a highly coordinated process involving changes in hormones, cytokines, adhesion molecules, enzymes, and growth factors [1,2]. In mammals, including pigs, the endometrium undergoes a transformation in response to the physiological changes triggered by ovarian hormones in different stages of the cycle to prepare for embryo attachment and implantation [3]. Early pregnancy in pigs is followed by a dynamic production of estrogens, prostaglandins, adhesion molecules, and immunological factors. Porcine embryos produce large amounts of estrogens, which enhance endometrial PGE2 production around days 11–12 of pregnancy, the period of maternal recognition of pregnancy [4,5]. With the trophoblast, rapid elongation and migration in the uterus, apposition, and attachment to the uterine surface epithelium occurs on days 16–18, initiating epitheliochorial placentation [6]. During the pre-attachment period, conceptuses undergo differentiation of trophectoderm for the secretion of an antiluteolytic or luteotrophic pregnancy recognition signal for maintenance of functional corpus luteum.

Litter size is one of the most economically important traits in pig production. However, reproductive traits in pigs are complex; from ovulation, fertilization, and implantation through to the birth of piglets, every step may affect litter size. Of these steps, implantation is the most important, since most embryonic deaths take place in this period. Approximately 45% of embryos for Large White and 21% for Meishan are lost between days 12 and 30 of pregnancy [7]. Periodic expression of many genes important for embryos occurs during implantation. Their proper and synchronized expression is essential for embryo survival and development. Systematic studies of transcriptome changes in the porcine endometrium during the time of implantation days 12, 14 [8,9,10], and days 15–16 [11] have been performed with DNA microarrays or RNA sequencing (RNA-Seq). However, transcriptomic analysis of the porcine endometrium during the whole implantation phase has not been performed. In this study, we use RNA-Seq to analyze the responses of the porcine endometrium to conceptus signals on day 12 (the time of maternal recognition), day 18 (conceptus attachment), and day 25 (embryo implantation) of pregnancy.

## 2. Experimental Section

### 2.1. Animals and Tissue Collection

Estrous behavior of sows was observed on every day. Sows exhibiting at least two estrous cycles of normal duration (21 days) were assigned randomly to three groups. Estrus occurred 24 h later (day 0), and the animals were inseminated twice with an interval of about 12 h. Nine sows were slaughtered at different stages of early pregnancy by electrical stunning: day 12 (*n* = 3), day 18 (*n* = 3), and day 25 (*n* = 3). The uteri were removed, and each uterine horn was subsequently flushed first with 10 mL and then with 50 mL of PBS buffer to collect conceptuses and uterine fluid. After flushing, uterine horns were opened longitudinally at the antimesometrial side. Several sections of the endometrium (not including the myometrium) were taken from the middle portion of the uterine horn of each sow on day 12. The samples of endometrium (peel off embryo) in attachment site (day 18) and implantation site (day 25) were collected. All tissue samples for isolation of RNA were immediately transferred to RNAlater (Invitrogen), incubated overnight at 4 °C, and stored at −80 °C until further use.

All experiments were approved by the Animal Ethics Committee, Shandong Academy of Agricultural Sciences, Jinan, China.

### 2.2. mRNA Library Construction and Sequencing

Total RNA was extracted using Trizol reagent (Invitrogen, Carlsbad, CA, USA) following the manufacturer’s procedures. The total RNA quantity and purity were analysis of Bioanalyzer 2100 and RNA 6000 Nano LabChip Kit (Agilent, Santa Clara, CA, USA) with RIN number > 8.0. Approximately 10 μg of total RNA representing a specific adipose type was subjected to isolate Poly (A) mRNA with poly-T oligo attached magnetic beads (Invitrogen).

Following purification, the mRNA is fragmented into small pieces using divalent cations under elevated temperature. Then the cleaved RNA fragments were reverse-transcribed to create the final cDNA library in accordance with the protocol for the mRNA-Seq sample preparation kit (Illumina, San Diego, CA, USA); the average insert size for the paired-end libraries was 300 bp (± 50 bp). Next we performed the paired-end sequencing on an Illumina Hiseq2000 at the LC-BIO (Hangzhou, China) following the vendor’s recommended protocol.

### 2.3. RNA-Seq Data Analysis

For each sample, sequenced reads were aligned to the UCSC [12] pig reference genome using the Tophat package, which initially removes a portion of the reads based on quality information accompanying each read and then maps the reads to the reference genome. Tophat allows multiple alignments per read (up to 20 by default) and a maximum of two mismatches when mapping the reads to the reference. Tophat builds a database of potential splice junctions and confirms these by comparing the previously unmapped reads against the database of putative junctions. The raw sequence data have been submitted to the NCBI Short Read Archive with accession number GSE73695.

### 2.4. Transcript Abundance Estimation and Differentially Expressed Testing

The aligned read files were processed by Cufflinks, which uses the normalized RNA-seq fragments counts to measure the relative abundances of the transcripts. The unit of measurement is Fragment Per Kilobase of exon per Million fragments mapped (FPKM). The reference GTF annotation file used in Cufflinks was downloaded from the UCSC database. Cufflink was used to *de novo* assemble the transcriptome at first; next, Cuffmerge was used to merge all transcripts of sample A and B to generate unique transcripts. The downloaded UCSC GTF file was passed to Cuffdiff along with the original alignment (SAM) files produced by Tophat. Cuffdiff re-estimates the abundance of the transcripts listed in the GTF file using alignments from the SAM file and concurrently tests for different expression. Only the comparisons with q value less than 0.01 and status marked as “OK” in the Cuffdiff output were regarded as showing differential expression.

### 2.5. Functional Enrichment Analysis

Assembled transcripts were converted to human orthologous genes, and the lists were submitted to the DAVID [13] web server [14] for enrichment analysis of the significant overrepresentation of GO biological processes (GO-BP), molecular function (GO-MF) terminologies, and KEGG-pathway category. In all tests, completely known genes were appointed as the background, and P-values (*i.e.*, EASE score), indicating significance of the overlap between various gene sets, were calculated using Benjamini-corrected modified Fisher’s exact test. Only GO-BP, GO-MF, or KEGG-pathway terms with a P-value less than 0.05 were considered significant and listed.

### 2.6. Quantitative Real-Time PCR

Quantitative RT-PCR (q-PCR) was used to measure the mRNA expression levels of eight representative indicators. Quantitative real-time PCR was performed on a Roche LightCycler^®^ 480 instrument (Roche, Penzberg, Germany) using SYBR^®^ Green Real-time PCR Master Mix (TaKaRa, Dalian, China). The total RNA samples used for qRT-PCR analyses are the same as the RNA-Seq acquired from the same nine sows. The PCR primer sequences are shown in Table 1. Gene expression levels were calculated using the ΔΔCt method and normalized using the geometric mean of expression levels of porcine ACTB and RN18S. The statistical difference in gene expression between the endometrium from different implantation periods was analyzed by t-test. Confirmed differences in gene expression were expressed as fold changes.

**Table 1:** Primer sequences for quantitative real-time RT-PCR.

Gene Symbol	Gene Name	Primer Sequence		Target Accession No.
*ACTB*	actin, beta	F:	CGAGCGCTTCCGGTGTCCAG	XM_003357928.1
		R:	GTGGTCCCGCCAGACAGCAC	
*RN18S*	18S ribosomal RNA	F:	GGGAGGAGGCTGACCGGGTT	NR_002170.3
		R:	ATACATGCCGACGGGCGCTG	
*FGF9*	fibroblast growth factor 9 (glia-activating factor)	F:	TTCCCAGGGGACCCGCAGTC	NM_213801.1
		R:	ATGCTGACCAAGCCCACGGC	
*IRF1*	interferon regulatory factor 1	F:	CCTTGTGCACCGTAGGCGGG	NM_001097413.1
		R:	GGCTTGCCAGGCCCCAAGAG	
*PTGES*	prostaglandin E synthase	F:	TGGTGAGCGGCCAGGTT	NM_001038631.1
		R:	TGGCCACTACGTACATCTTGATG	
*OSTN*	osteocrin	F:	CCCCTGGACAGACTCTCAGCAGG	NM_001098597.1
		R:	GCCTCTGGAATTTGGAAGCCGGT	
*S100A9*	S100 calcium binding protein A9	F:	ACCACATCCTGGAAGACCTG	NM_001177906.1
		R:	TCCTCGTGAGAAGCTACCGT	
*STAT1*	signal transducer and activator of transcription 1, 91 kDa	F:	AAATGCCGGCGCCAGAACCA	NM_213769.1
		R:	CGGGAGCTGGCTGACGTTGG	
*STC1*	stanniocalcin 1	F:	ACACGCACCAGCGAGCTGAC	NM_001103212.1
		R:	GCTGTGAACACCTCGCCCCC	

## 3. Results

### 3.1. RNA-Seq Data for the Endometrium on Days 12, 18, and 25 of Pregnancy

From 34,275,994 to 124,850,826 raw reads and from 33,688,562 to 122,914,990 quality-filtered reads were found per sample. Using the Tophat package [15], 55.84%–77.99% reads were mapped (< 2 mismatches) to the UCSC pig reference genome, 51.42%–72.25% reads had a unique genomic location (Table 2). Then, Cufflinks [16] was used to *de novo* assemble the porcine endometrium transcriptome, 45,828 transcripts were assembled and 20,310 genes were named in the UCSC pig genome database.

**Table 2:** Summary of RNA-Seq alignment.

Sample	Raw Reads	Clean Reads	Clean Data	Mapped Reads	Unique Mapped Reads	Multi Mapped Reads	Pair-End Mapped Reads
D12-01	67169826	66077622	66077622	49833482	46142686	3690796	23127536
D12-02	41784360	39324942	39324942	29499855	27337499	2162356	13706839
D12-03	34275994	33688562	33688562	25394236	23553198	1841038	11998456
D18-01	41684596	40824568	40824568	26668182	24658656	2009526	12636036
D18-02	40426950	39987798	39987798	22327827	20562051	1765776	10550537
D18-03	44795678	44415892	44415892	28210350	26213892	1996458	13001074
D25-01	50047464	49009334	49009334	37099086	33754041	3345045	17284406
D25-02	84890198	83601216	83601216	64826626	57999868	6826758	30054104
D25-03	124850826	122914990	122914990	95857938	88801603	7056335	43719905

**Figure 1:**
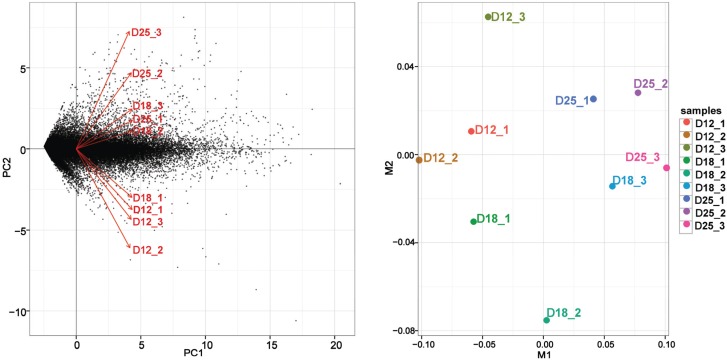
Principal component analysis (PCA) and multidimensional scaling (MDS) results of RNA-Seq data of endometrium on days 12, 18, and 25 of pregnancy.

The RNA-Seq alignment is similar to all samples collected from the endometrium on day 18 of pregnancy. However, the alignment is quite variable between samples collected on day 12 and day 24 of pregnancy. This kind of variability is possibly the result of different sampling sites or implantation of the endometrium. On day 12 of pregnancy, the samples of endometrium were taken from the antimesometrial side and the middle portion of each uterine horn of each sow; there may be a difference between the sampling sites of different pigs due to the embryo not having attached to the uterine wall, and the RNA-seq alignment was variable in this stage. Because the conceptus attachment arises from about day 18 of pregnancy, the status of the endometrium (attachment site) is similar. However, the time of implantation of a fertilized egg may be different due to the diversity among conceptuses, and this results in differences in the samples collected on day 25. Therefore, there is more variability of RNA-seq alignment on day 25 than on day 18 of pregnancy (Figure 1).

### 3.2. Gene Expression Variations between the Different Implantation Stages

**Figure 2:**
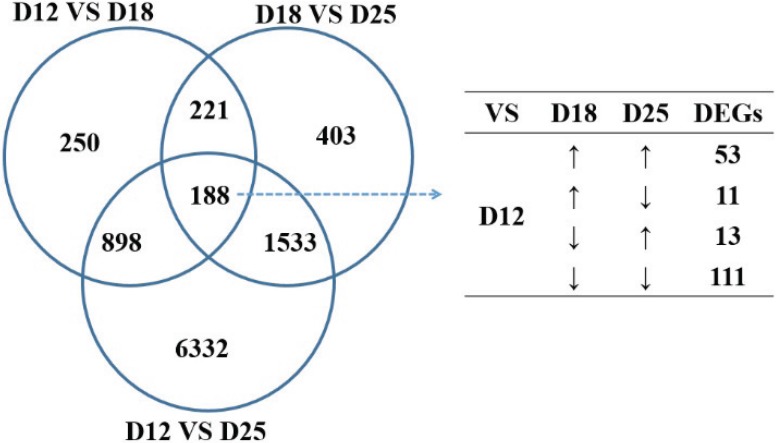
Differentially expressed genes (DEGs) of the endometrium for the three periods of pregnancy.

FPKM of each gene was processed by Cufflinks, and Cuffmerge was used to merge all transcripts of sample A and B to generate unique transcripts. To identify the differentially expressed genes during different implantation stages, the comparisons were analyzed between day 12 and 18, day 18 and 25, and day 12 and 25, respectively. The amount of differentially expressed genes (DEGs) between day 12 and 25 was the most (8951 differentially expressed genes), followed by between day 18 and 25 (Table 3). A total of 188 transcripts were expressed differentially among the three stages of pregnancy (Figure 2), of which 60 genes were upregulated (*i.e.*, DPPA5, OSTN, PPARG, TIMP1, *etc.*) and 38 genes downregulated with the stage (*i.e.*, STC1, SATB1, *etc.*). Sixty-four of these were significantly increased on day 18 with regard to days 12 and 25 (*i.e.*, FETUB, IFNG, ITGB2, ITGA4, *etc.*), and 26 were significantly decreased on day 18 compared with days 12 and 25 (*i.e.*, SELL, MMP7, *etc.*).

**Table 3:** Differentially expressed genes (DEGs) of the endometrium in the three periods of pregnancy.

Sample	TB Gene Number	DEGs	Upregulation	Downregulation
D12 *vs.* D18	45828	1557	708	849
D12 *vs.* D25		8951	4999	3952
D18 *vs.* D25		2345	1166	1179

### 3.3. Gene Ontology (GO) and Genomes (KEGG) Ontology (KO) Classification (Up Arrow Indicates Upregulation, and Down Arrow Indicates Downregulation)

**Figure 3:**
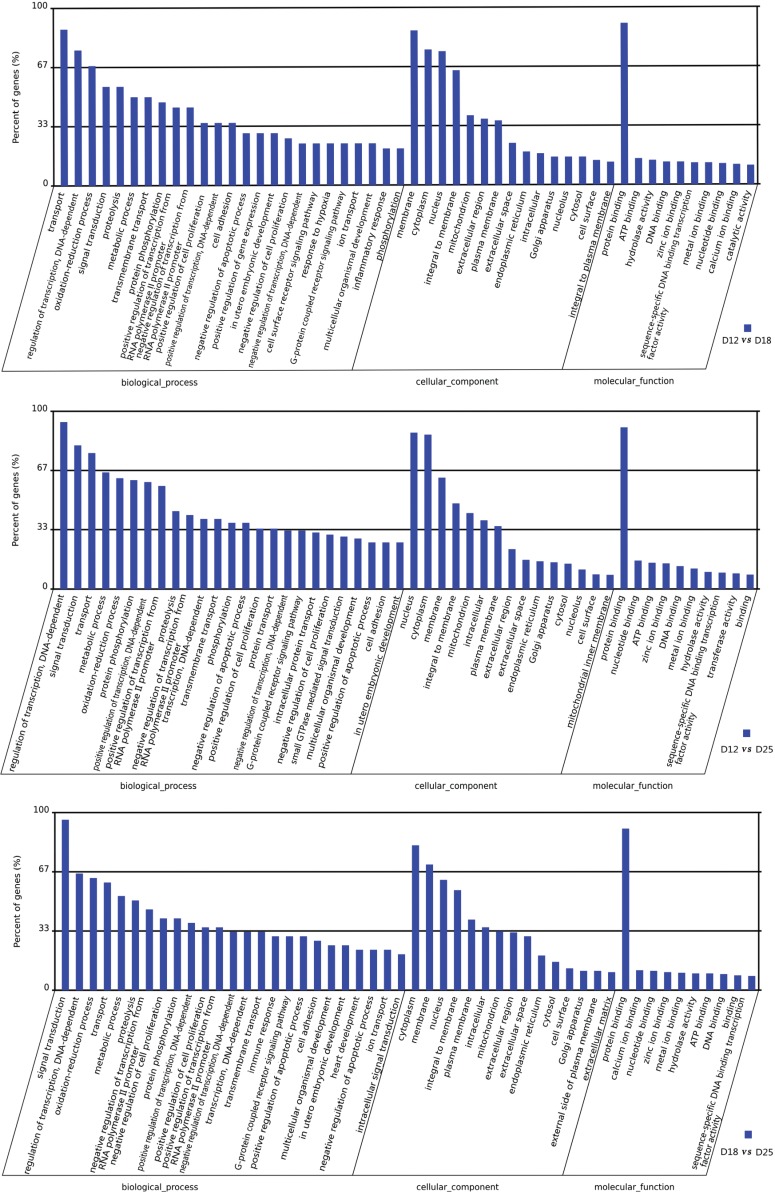
GO categories of the transcripts expressed differently between the three periods (days 12, 18, and 25) of pregnancy. Transcripts were annotated in three categories: cellular components, molecular functions, and biological processes.

There are significant differences in expression between days 12 and 18 of pregnancy. A total of 1557 genes, of which 708 were upregulated and 849 downregulated, were identified on day 12 *vs.* 18. According to the GO enrichment analyses, in the category of molecular function (MF), the most genes clustered in “protein binding” (Figure 3). In the category of biological process (BP), the DEGs clustered in “transport”, “regulation of transcription, DNA-dependent” “oxidation-reduction process”, “signal transduction”, *etc.* In the category of cellular components, the differentially expressed genes were mainly related to “membrane”, “cytoplasm”, “nucleus”, and “integral to membrane”.

A total of 8951 and 2345 genes were differentially expressed in the endometrium between D12 *vs.* D18 and D18 *vs.* 25 (Table 3), respectively. As a comparison between D12 *vs.* D18, the most genes between D12 *vs.* D25 and D18 *vs.* D25 were related to “protein binding” in the category of MF. On the other hand, the DEGs clustered in “transport”, “regulation of transcription, DNA-dependent”, and “signal transduction” are in the category of BP.

The biological pathways of significant DEGs in different periods of implantation are shown in Table 4. According to the KEGG database, the most significant DEGs between day 12 and 18, and day 18 and 25, were clustered in the “cytokine-cytokine receptor interaction”, “complement and coagulation cascades”, and “focal adhesion” pathways. The DEGs between day 12 and 25 were enriched in the “protein processing in endoplasmic reticulum”, “cell cycle”, “oxidative phosphorylation”, “phagosome”, “ribosome biogenesis in eukaryotes”, “axon guidance”, “Wnt signaling”, “focal adhesion”, and “complement and coagulation cascades” pathways, *etc.*

**Table 4:** The most significant DEGs biological pathways examined with the KEGG database (*p* ≤ 0.01).

		Pathway ID	Pathway Name	Gene Number	*p*-Value	Gene List *
Day 12 *vs.* Day 18	1.	ssc04060	Cytokine–cytokine receptor interaction	13	1.79 × 10^−4^	***ACVR1***, ***ACVR1B***, ***FIGF***, ***MET***, *CCR7*, *CSF1R*, *CSF2RB*, *CXCL14*, *CXCR3*, *IFNGR1*, *IL10RA*, *IL21R*, *IL2RG*
	2.	ssc04610	Complement and coagulation cascades	10	2.26 × 10^−7^	***C7***, ***DF***, ***F5***, *C1QA*, *C2*, *CFH*, *F10*, *FGB*, *KNG1*, *PLAUR*
	3.	ssc04510	Focal adhesion	8	4.19 × 10^−5^	***COL5A3***, ***ITGB8***, *COL11A1*, *ITGA4*, *LAMA3*, *MYL7*, *PARVG*, *SPP1*
	4.	ssc04110	Cell cycle	8	7.39 × 10^−4^	***CDKN2B***, ***CDKN2B***, ***PLS1***, ***SMAD2***, *CDC2*, *CDC6*, *SKP2*, *WEE1*
	5.	ssc00270	Cysteine and methionine metabolism	6	1.18 × 10^−6^	***CDO1***, *AHCY*, *AMD1*, *DNMT3B*, *MAT1A*, *SMS*
	6.	ssc04142	Lysosome	6	1.66 × 10^−3^	***AP1B1***, *CTSC*, *CTSH*, *CTSZ*, *DNASE2*, *NPC2*
	7.	ssc04360	Axon guidance	6	4.97 × 10^−3^	***EFNA1***, ***SEMA6A***, *EPHA2*, *SEMA3F*, *SEMA4A*, *UNC5B*
	8.	ssc00190	Oxidative phosphorylation	6	8.96 × 10^−3^	***ATP6V1C2***, *ATP5G1*, *ATP6V1G3*, *NDUFA10*, *NDUFS3*, *UQCRC1*
	9.	ssc04514	Cell adhesion molecules (CAMs)	6	8.96 × 10^−3^	***SELL***, *CD2*, *CD4*, *CD8A*, *ITGAL*, *PTPRC*
	10.	ssc00860	Porphyrin and chlorophyll metabolism	5	6.97 × 10^−5^	***CP***, *CPOX*, *EPRS*, *HMBS*, *UROD*
	11.	ssc00512	Mucin type O-Glycan biosynthesis	5	1.98 × 10^−4^	***GALNT11***, ***GALNTL5***, ***GCNT1***, *B4GALT5*, *GALNT9*
	12.	ssc04115	p53 signaling pathway	4	1.98 × 10^−4^	***SESN3***, *CYCS*, *GTSE1*, *SERPINB5*
	13.	ssc04650	Natural killer cell mediated cytotoxicity	4	5.28 × 10^−4^	*CD244*, *CD48*, *FCER1G*, *PRF1*
	14.	ssc03320	PPAR signaling pathway	4	5.00 × 10^−3^	***CD36***, *APOA1*, *FABP5*, *PPARG*
	15.	ssc02010	ABC transporters	4	5.95 × 10^−3^	***ABCA1***, ***ABCB10***, *ABCC2*, *CFTR*
	16.	ssc04614	Renin–angiotensin system	3	1.88 × 10^−5^	***ACE2***, ***LNPEP***, ***MME***
	17.	ssc04350	TGF-beta signaling pathway	3	3.72 × 10^−4^	***FST***, *ID1*, *ID2*
	18.	ssc05010	Alzheimer’s disease	2	6.84 × 10^−3^	*APOE*, *BACE2*
Day 12 *vs.* Day 25	1.	ssc04141	Protein processing in endoplasmic reticulum	30	6.70 × 10^−6^	***ATF6B***, ***BAK1***, ***DNAJB2***, ***HSPA4L***, ***UBXN6***, *CALR*, *CRYAB*, *DERL3*, *DNAJA1*, *DNAJB11*, *DNAJB12*, *DNAJC10*, *ERLEC1*, *ERP29*, *GRP-58*, *HSP90AA1*, *HSP90B1*, *HSPH1*, *LMAN1*, *NGLY1*, *PDIA4*, *PLAA*, *SAR1A*, *SAR1B, SEC23B, SEC31A, SSR1, SSR4, TRAM1, VIMP*
	2.	ssc04110	Cell cycle	29	1.88 × 10^−9^	***ANAPC5***, ***ATM***, ***CDC14A***, ***CDKN2B***, ***EP300***, ***FZR1***, ***PLS1***, ***SMAD2***, *ATM*, *BUB3*, *CCNB2*, *CDC2*, *CDC23*, *CDC25C*, *CDC25C*, *CDC6*, *CDK6*, *CDKN2C*, *E2F1*, *E2F3*, *HDAC2*, *MAD2L1*, *ORC3*, *PTTG1*, *SKP2*, *SMAD4*, *TTK*, *WEE1*, *YWHAH*
	3.	ssc00190	Oxidative phosphorylation	25	5.06 × 10^−6^	***ATP6V0D2***, ***TCIRG1***, *ATP5A1*, *ATP5G1*, *ATP5G3*, *ATP6V0D1*, *ATP6V0E1*, *ATP6V1C1*, *ATP6V1E1*, *ATP6V1G3*, *ATP6V1H*, *COX5B*, *COX7C*, *NDUFA5*, *NDUFAB1*, *NDUFB3*, *NDUFB5*, *NDUFB6*, *NDUFS1*, *NDUFS3*, *NDUFS4*, *NDUFS5*, *NDUFV1*, *NDUFV2*, *UQCRH*
	4.	ssc04145	Phagosome	19	5.71 × 10^−4^	***CD61***, ***ITGAV***, ***MRC2***, ***SCARB1***, ***SFTPD***, ***SLA***, ***SLA-DMA***, ***TUBA4A***, *CH242-21O2.1*, *DYNC1LI1*, *FCGR1A*, *ITGA5*, *ITGB1*, *MRC2*, *MSR1*, *NCF2*, *THBS1*, *THBS3*, *TLR2*
	5.	ssc03008	Ribosome biogenesis in eukaryotes	18	1.49 ×10 ^−4^	***RPP25L***, ***XRN1***, *DKC1*, *GNL3*, *LSG1*, *MPHOSPH10*, *NHP2L1*, *NXT2*, *POP5*, *RAN*, *REXO2*, *RIOK1*, *RPP30*, *RPP40*, *SBDS*, *UTP18*, *UTP6*, *WDR75*
	6.	ssc04360	Axon guidance	17	5.74 × 10^−3^	***ABLIM1***, ***EFNA1***, ***EPHB4***, ***FYN***, ***L1CAM***, ***LIMK2***, ***SEMA3G***, ***SEMA6A***, ***SEMA6C***, ***SEMA6D***, *CFL2*, *EPHA2*, *EPHA4*, *EPHB2*, *SEMA3F*, *SEMA4F*, *UNC5B*
	7.	ssc04310	Wnt signaling pathway	16	4.82 × 10^−3^	***CHD8***, ***CSNK1E***, ***DVL1***, ***FZD10***, ***FZD5***, ***NFATC4***, ***SOX17***, ***TCF7L2***, ***VANGL1***, ***VANGL2***, *CACYBP*, *FOSL1*, *SFRP2*, *SFRP5*, *TCF7L1*, *WNT2B*
	8.	ssc04510	Focal adhesion	15	1.66 × 10^−3^	***ITGA3***, ***ITGA6***, ***ITGB4***, ***ITGB8***, ***VWF***, *COL11A1*, *COL5A2*, *FN1*, *ITGA1*, *ITGA11*, *ITGA4*, *ITGA8*, *LAMA3*, *MYL7*, *VCL*
	9.	ssc04610	Complement and coagulation cascades	13	5.65 × 10^−3^	***DF***, ***F5***, ***SERPINC1***, *C2*, *C7*, *CFH*, *CFI*, *FGB*, *MASP1*, *PLAU*, *PLAUR*, *SERPINA5*, *TFPI*
	10.	ssc04115	p53 signaling pathway	9	4.02 × 10^−4^	***CCNG2***, ***SESN3***, ***SHISA5***, *BID*, *CYCS*, *EI24*, *GTSE1*, *SERPINB5*, *TP53I3*
	11.	ssc04530	Tight junction	8	6.02 × 10^−3^	***CGN***, ***EPB41L1***, ***MAGI3***, ***MPP5***, *AMOTL1*, *CRB3*, *EPB41L3*, *MYH1*
	12.	ssc00860	Porphyrin and chlorophyll metabolism	8	2.04 × 10^−3^	***HMOX2***, *BLVRA*, *CPOX*, *EPRS*, *HMBS*, *MMAB*, *UROD*, *UROS*
	13.	ssc00480	Glutathione metabolism	7	2.04 × 10^-3^	***GCLC***, ***GSTM3***, *GCLM*, *GPX2*, *GSTT1*, *MGST3*, *TXNDC12*
	14.	ssc00260	Glycine, serine and threonine metabolism	7	6.02 × 10^−3^	***CHDH***, ***GATM***, ***GLYCTK***, ***GNMT***, ***PHGDH***, ***PSAT1***, ***PSPH***
	15.	ssc00512	Mucin type O-Glycan biosynthesis	7	9.44 × 10^−3^	***GALNT11***, ***GALNTL5***, ***GCNT1***, *B4GALT5*, *GALNT10*, *GALNT3*, *GALNT9*
	16.	ssc00100	Steroid biosynthesis	7	2.68 × 10^−4^	***LIPA***, *CYP51*, *HSD17B7*, *NSDHL*, *SC4MOL*, *SQLE*, *TM7SF2*
	17.	ssc05322	Systemic lupus erythematosus	7	1.70 × 10^−3^	***H2AFV***, ***TRIM21***, *H2AFZ*, *H3F3A*, *H3F3C*, *HIST2H2AC*, *SSB*
	18.	ssc00520	Amino sugar and nucleotide sugar metabolism	7	6.10 × 10^−3^	***CYB5R3***, ***GNPDA1***, *CHI3L1*, *CMAS*, *GNPDA2*, *NANS*, *UAP1*
	19.	ssc03060	Protein export	7	6.10 × 10^−3^	*HSPA5*, *SEC11C*, *SEC61B*, *SEC61G*, *SPCS1*, *SPCS3*, *SRPRB*
	20.	ssc05010	Alzheimer’s disease	6	4.90 × 10^−4^	***ADAM10***, ***CDK5R1***, ***SNCA***, *APOE*, *BACE2*, *IDE*
	21.	ssc04972	Pancreatic secretion	4	9.37 × 10^−3^	***RAB27B***, ***RAB3D***, ***TPCN2***, *RAB8A*
	22.	ssc00740	Riboflavin metabolism	4	6.14 × 10^−3^	***FLAD1***, ***RFK***, *ACP1*, *ACP6*
Day 18 *vs.* Day 25	1.	ssc04060	Cytokine–cytokine receptor interaction	19	7.75 × 10^−6^	***CCR7***, ***CD27***, ***CD40***, ***CSF1R***, ***CXCL10***, ***CXCL16***, ***CXCR3***, ***FLT3LG***, ***IFNE***, ***IL10RA***, ***IL21R***, ***IL2RG***, ***LIFR***, ***LTB***, ***MET***, ***TNFRSF21***, *CSF3*, *IL13RA1*, *IL18*
	2.	ssc04145	Phagosome	13	3.31 × 10^−7^	***CD61***, ***DMB***, ***SLA***, ***SLA-DMA***, ***TUBA4A***, *CD209*, *CH242-21O2.1*, *ITGB1*, *MRC2*, *MSR1*, *THBS1*, *THBS2*, *THBS3*
	3.	ssc00190	Oxidative phosphorylation	12	1.18 × 10^−5^	***ATP6V0C***, ***ATP6V0D2***, ***NDUFS6***, ***TCIRG1***, *ATP6V1C2*, *ATP6V1E1*, *NDUFA5*, *NDUFB3*, *NDUFB6*, *NDUFS4*, *NDUFS5*, *NDUFV2*
	4.	ssc04610	Complement and coagulation cascades	11	1.12 × 10^−6^	***C1QA***, ***C8G***, ***DF***, ***FGB***, ***KNG1***, ***SERPINA1***, ***SERPINA5***, ***SERPINC1***, *C7*, *CFH*, *CFI*
	5.	ssc04510	Focal adhesion	10	1.96 × 10^−5^	***COL11A1***, ***ITGA3***, ***ITGA4***, ***LAMB3***, ***PARVG***, *COL5A2*, *ITGA11*, *ITGA8*, *MYL7*, *VCL*
	6.	ssc04514	Cell adhesion molecules (CAMs)	8	5.26 × 10^−3^	***CD8A***, ***CLDN8***, ***ITGAL***, ***PTPRC***, *PECAM1*, *SELL*, *VCAM1*, *VCAN*
	7.	ssc00260	Glycine, serine, and threonine metabolism	5	9.15 × 10^−5^	***GLYCTK***, ***GNMT***, ***PHGDH***, ***PSAT1***, *AOC3*
	8.	ssc03320	PPAR signaling pathway	5	3.57 × 10^−3^	***APOA1***, ***APOA2***, ***APOC3***, ***PPARD***, *PPARG*
	9.	ssc04270	Vascular smooth muscle contraction	4	2.04 × 10^−4^	*ACTA2*, *CALD1*, *KCNMB1*, *PPP1R12B*
	10.	ssc04650	Natural killer cell-mediated cytotoxicity	4	2.43 × 10^−3^	***CD3Z***, ***CD48***, ***PRF1***, ***ZAP70***
	11.	ssc00480	Glutathione metabolism	4	4.53 × 10^−3^	*GCLC*, *GCLM*, *GPX2*, *TXNDC12*
	12.	ssc00590	Arachidonic acid metabolism	4	7.58 × 10^−3^	*ALOX12*, *ALOX15B*, *LTA4H*, *PTGIS*
	13.	ssc00340	Histidine metabolism	3	4.01 × 10^−3^	***HAL***, ***HDC***, ***UROC1***
	14.	ssc00100	Steroid biosynthesis	3	8.91 × 10^−3^	*CYP51*, *HSD17B7*, *SC4MOL*

* Upregulated genes are marked by bold font and downregulated genes are marked by regular font.

Three genes (CDKN2B, ATM, and CDC25C) are duplicated in Table 4. CDKN2B is in bold (upregulated) and included twice as a DEG in the cell cycle pathway of day 12 *vs.* day 18 of pregnancy; this is because alternative splicing events happen in this gene location (chr1:223955644-223965842), and these two alternative transcriptions of CDKN2B are DEG on day 12 and day 18. In the same way, the gene CDC25C is in regular font (downregulated) and included twice as a DEG in the cell cycle pathway of day 12 *vs.* day 25 of pregnancy (alternative splicing on chr2:145985624-146023775). However, the gene ATM is in both bold and regular font (Table 4) when comparing DEGs obtained from endometrium on day 12 *vs.* day 25; two variable transcripts (chr9:40925894-40945439 and 40746502-40833540) are present in different expression patterns, the transcript of ATM chr9:40925894-40945439 upregulated and chr9:40746502-40833540 downregulated on day 25 as compared to day 12.

### 3.4. Validation of the RNA-Seq Data

To validate the RNA-Seq data, eight genes were selected for q-PCR. The samples used for qRT-PCR analyses are the same as RNA-Seq; they were acquired from the same nine sows and thus result in a good agreement between the two techniques, as shown in Table 5. As mentioned in the results (Section 3.1), the RNA-seq data are quite variable between samples collected on days 12 and 24 of pregnancy. This kind of variability is possibly the result of different sampling sites or implantation of the endometrium. Therefore biologically reproducible studies are necessary to more accurate results. The genes selected for real-time PCR (Table 3) are involved in the following aspects of the regulation of early pregnancy: fibroblast growth (FGF9), immune response (IRF1), adhesion (OSTN), prostaglandin synthase (PTGES), signal transducer and activator of transcription (STAT1), and implantation marker (STC1).

**Table 5:** Results of selected gene expression validation with quantitative real-time PCR.

Gene	*vs.*	Fold-Change		*p*-Value		Correlation
		RNA-Seq	Real-time PCR	RNA-Seq	Real-time PCR	
*FGF9*	D12 *vs.* D18	1.01	0.89	0.9076	0.7736	0.8825
	D12 *vs.* D25	2.92	2.87	< 0.0001	< 0.0001	0.9939
	D18 *vs.* D25	2.82	3.32	0.0002	< 0.0001	0.9692
*IRF1*	D12 *vs.* D18	1.16	1.83	0.7325	0.2655	0.9800
	D12 *vs.* D25	5.62	7.54	< 0.0001	< 0.0001	0.8467
	D18 *vs.* D25	4.72	4.23	0.0005	0.0136	0.9092
*OSTN*	D12 *vs.* D18	−17.40	−15.82	< 0.0001	< 0.0001	0.9732
	D12 *vs.* D25	−530.57	−1015.42	< 0.0001	< 0.0001	0.7383
	D18 *vs.* D25	−30.93	−63.69	0.0003	0.0009	0.9799
*PTGES*	D12 *vs.* D18	−3.45	−5.37	< 0.0001	< 0.0001	0.9606
	D12 *vs.* D25	−4.05	−13.64	< 0.0001	< 0.0001	0.9807
	D18 *vs.* D25	−1.15	−2.55	0.2223	0.0912	0.9201
*S100A9*	D12 *vs.* D18	610.76	329.82	< 0.0001	< 0.0001	0.7967
	D12 *vs.* D25	372.60	266.37	< 0.0001	0.0002	0.8823
	D18 *vs.* D25	0.60	0.83	0.0685	0.0502	0.9734
*STAT1*	D12 *vs.* D18	0.91	0.62	0.9542	0.3827	0.9987
	D12 *vs.* D25	1.98	0.87	0.0001	0.0106	0.9336
	D18 *vs.* D25	2.22	1.19	< 0.0001	0.0114	0.9725
*STC1*	D12 *vs.* D18	9.40	8.39	< 0.0001	< 0.0001	0.9480
	D12 *vs.* D25	51.34	38.83	< 0.0001	< 0.0001	0.8834
	D18 *vs.* D25	5.91	4.43	< 0.0001	0.0057	0.9508

## 4. Discussion

In pigs, maternal recognition of pregnancy takes place around day 11 and implantation occurs on days 12–26. In the present study, we collected porcine endometrial tissue samples on day 12 (the time of maternal recognition), day 18 (conceptus attachment), and day 25 (embryo implantation) of pregnancy. To ensure consistency between samples, the myometrium was peeled off. On day 12 of gestation, conceptuses are already elongated but not attached to the uterine wall [17]; the samples of endometrium were taken from the middle portion of each uterine horn and at the antimesometrial side. The endometrial samples on days 18 and 25 of pregnancy were collected from the implantation zones (with the embryo peeled off first) of pregnant gilts.

The RNA-Seq data were analyzed to compare different stages of implantation (days 12, 18, and 25). The results revealed that there are comprehensive transcriptome changes in response to the implantation process. DEGs were highest between days 12 and 25, followed by between days 18 and 25. The variance in the number of DEGs indicates that the cell activity and gene expression changes in the endometrium were dramatic on day 25 *vs.* 12. It is mostly likely that the elongating porcine embryos finish their migration through the uterus and start to attach to the luminal epithelium of the endometrium on day 12 [18], while on day 25 the embryo had basically completed the implantation process. As we know, successful implantation requires synchrony between uterus and embryo, and implantation happens during the “implantation window”. Periodic expression of many genes important for embryos occurs during implantation. This result agrees with findings from a proteomics study of uterine secretions from days 10 and 13 of the estrous cycle and pregnancy [19,20], in which those authors found more differences in the protein amounts over time than were attributed to pregnancy status.

Transcriptome changes in the porcine endometrium during days 12, 14 [8,9,10] and days 15–16 [11] have been performed with DNA microarrays or RNA-Seq. These studies have focused on the DEGs between pregnancy and the corresponding day of the estrous cycle (or non-pregnancy), and the periods are mainly in the early stages of implantation (days 12–16). In the present study, we obtained the transcriptome profile of the endometrium in the whole embryo implantation process, and the genes’ expression was compared between = early, mid-, and late implantation, respectively. A large number of DEGs were filtered; results indicated that uterine endometrial responses to implantation are complex. In order to achieve a state of endometrial receptivity, a lot of endometrial physiology and cell activities happened in the porcine endometrium during implantation, which is regulated by numerous genes, and the time and space of gene expression is critical for proper embryo–maternal crosstalk and successful embryo implantation. Systematic studies of porcine endometrium are essential for embryo survival and development in early pregnancy.

Early pregnancy is accompanied by many immune reactions simultaneously occurring in the uterus [21,22]. In contrast to day 25 of pregnancy, there is a particularly high overrepresentation of genes associated with the immune response on days 12 and 18, including CD27, CD3D, CD3E, CD3Z, CD40, CD48, CD5, CD6, CD61, CD8A, and ZAP70. In this study, the interesting result is about S100A9, the expression level of which is huge on day 12 of pregnancy, but decreases significantly by days 18 and 25 (Table 5). S100A9 is an inflammatory protein and it exhibits a cytokine-like function, enhancing leukocyte recruitment to the inflammatory site. The expression of S100A9 was associated with early pregnancy loss [23,24]. Immune response and cell-adhesion factors expressed in the endometrium on days 15–16 ensure proper embryo–maternal crosstalk [11]. The main adhesion factors appear in luminal epithelial cells during the phase of endometrial receptivity [25]. Although the adhesion of the embryos to the endometrium can be regulated at the gene expression level, the global endometrial profile of genes encoding adhesion factors was previously unknown. We found many genes involved in the regulation of cell adhesion in porcine pregnancy, including “Focal adhesion” and the “Cell adhesion molecules (CAMs)” pathway, and for the first time we obtained a complete biological pathway list of upregulated and downregulated genes encoding adhesion molecules in the porcine endometrium during early, mid-, and late implantation (Table 4).

According to the results of KEGG analysis, multiple genes related to the pathway “cytokine-cytokine receptor interaction” were significantly overexpressed in the endometrium on day 18 of gestation *vs.* days 12 and 25, including CCR7, CSF1R, CXCR3, IL10RA, and IL2RG (Table 3). Its function may be to promote correct communication between the endometrium and the developing conceptus, and to achieve the readiness of uterus for implantation. The uterus has greater receptivity in mid-implantation than in the early and late periods. Many studies indicate that cytokines are important to embryo development and successful implantation. Not only do certain cytokines have completely different effects on implantation—for example, CSF promotes implantation and early development while TNF is deleterious—but the level of the cytokine present is also important. In some cases, absence of a cytokine, for example LIF, is associated with failure of implantation while excess concentrations of a cytokine, for example CSF, can abort an early pregnancy [26,27,28].

In pigs, fertility rates are generally very high but the early embryonic loss that occurs on days 12–30 of gestation critically affects the potential litter size. Temporal changes that take place in the endometrial environment during the period of early pregnancy in pigs play an important role in embryonic survival and successful pregnancy. Systematic studies of the molecular changes associated with these processes will pave the way for an understanding of endometrial functions and help to increase embryo survival. In the present study, we obtained comprehensive transcriptomic profiles of the porcine endometrium during pregnancy, but one limitation is surely the analysis of endometrium between different implantation stages of pregnancy, not compared with non-pregnancy. This may result in partial genes, which expressed continuously and no significant changes during implantation, were failed to discover. Nevertheless, the gene expression data generated in the present study represent a rich resource for further targeted studies of genes and pathways potentially involved in the regulation of this process.

## 5. Conclusions

The present study identified comprehensive transcriptome changes in the porcine endometrium in early, mid-, and late implantation. Several biological processes and pathways potentially involved in embryo implantation in the early pregnancy of sows were identified by bioinformatics analysis, especially cell proliferation, the regulation of the immune response, the interaction of cytokine receptors, and cell adhesion. This comprehensive identification of transcriptomic changes in the porcine endometrium could be a foundation for targeted studies of genes and pathways that are potentially involved in abnormal endometrial receptivity and embryo loss in early pregnancy.
